# Neutrophil Aggregation in a Relapsed Case of Idiopathic Thrombocytopenic Purpura: A Case Report

**DOI:** 10.7759/cureus.79076

**Published:** 2025-02-16

**Authors:** Maher Aljohani, Asma Alfetayeh, Turki Alwasaidi

**Affiliations:** 1 Pathology, Prince Mohammed Bin Abdul Aziz Hospital (PMBAH) Ministry of National Guard Health Affairs (MNGHA), Madinah, SAU; 2 Hematology, Prince Mohammed Bin Abdul Aziz Hospital (PMBAH) Ministry of National Guard Health Affairs (MNGHA), Madinah, SAU; 3 Medicine, Prince Mohammed Bin Abdul Aziz Hospital (PMBAH) Ministry of National Guard Health Affairs (MNGHA), Madinah, SAU

**Keywords:** autoimmune hemolytic anemia (aiha), complete blood count (cbc), coronavirus disease 2019 (covid-19), edta ethylenediamine tetra acetic acid, granulocyte colony-stimulating factor (g-csf), immune mediated thrombocytopenic purpura (itp), plt : platelets, proton-pump inhibitors (ppi)

## Abstract

This case report highlights a rare instance of pseudo-neutropenia involving in vitro alterations in neutrophils in a 67-year-old male with relapsed idiopathic thrombocytopenic purpura (ITP). Pseudo-neutropenia, also known as spurious neutropenia, refers to in vitro changes in neutrophil counts that do not accurately reflect the patient’s clinical condition. This patient also exhibited pseudo-thrombocytopenia and cold agglutination in vitro. This phenomenon is often induced by the anticoagulant ethylenediaminetetraacetic acid (EDTA) or the presence of cold agglutinins or combined with a medication reaction.

## Introduction

Pseudo-neutropenia is a laboratory artifact characterized by the apparent reduction in neutrophil counts, which arises from sample handling discrepancies or specific patient conditions, rather than a genuine decrease in neutrophil levels [1-4]. This phenomenon is attributed to in vitro neutrophil agglutination, often induced by the anticoagulant ethylenediaminetetraacetic acid (EDTA) or the presence of cold agglutinins. Distinguishing pseudo-neutropenia from true neutropenia is crucial to avoid unnecessary medical interventions [5-7].

In instances where laboratory results appear discordant with the clinical presentation of a patient, it is advisable to consider retesting or employing additional diagnostic approaches. Pseudo-neutropenia may occur due to leukocyte clumping or improper storage of blood samples. Although the precise mechanism underlying neutrophil agglutination remains unclear, it can be detected through the examination of a peripheral blood smear for leukocyte aggregates [8-9]. In the context described, pseudo-neutropenia does not carry any pathophysiological implications. However, it may inadvertently prompt a range of unwarranted medical interventions, including antibiotic therapy, administration of granulocyte-colony stimulating factor (G-CSF), hospitalization, or bone marrow biopsy, all of which may be unnecessary or inappropriate [10-12]. This case report highlights a rare instance of pseudo-neutropenia involving in vitro alterations in neutrophils in a 67-year-old male with relapsed idiopathic thrombocytopenic purpura (ITP). Pseudo-neutropenia, also known as spurious neutropenia, refers to in vitro changes in neutrophil counts that may not accurately represent the patient’s clinical condition.

## Case presentation

A 67-year-old male was first diagnosed with COVID-19 in June 2020. After three months, the patient started showing symptoms of rush over his limbs and epistaxis and was diagnosed with ITP/autoimmune hemolytic anemia (AIHA). He was treated with a six-unit platelet transfusion. He was discharged on tranexamic acid (500 mg) and prednisolone (55 mg). The patient returned for a follow-up one month later, in October 2020, as a case of ITP with the same complaints but no evidence of AIHA. He continued treatment with prednisolone (50 mg for five days, then tapered by 5 mg every three days), folic acid (5 mg), tranexamic acid (500 mg), and vitamin D₃ (50,000 IU per week). At that time, a peripheral blood smear from the EDTA tube was done; the test indicated marked platelet clumping. A sodium (Na) citrate tube was requested, revealing a few small clumps and showing mild thrombocytopenia on manual estimation (PLT: 106 × 10⁹/L) (Table 1). Consequently, a bone marrow test was performed, and the morphological findings were consistent with pseudo-thrombocytopenia as the current cause of the low platelet count. The cytogenetic results were normal. After that, the patient remained asymptomatic until a routine follow-up visit in June 2021. Blood tests revealed his first relapse, despite the absence of symptoms. The CBC indicated a critically low platelet count (PLT: 10 × 10⁹/L), with no evidence of platelet clumping (Table 1).

**Table 1:** Platelet (PLT) count of the patient during clinic visits.

Date	PLT
October 14, 2020	106 x 10^9^/L
January 24, 2021	157 x 10^9^/L
June 13, 2021	10 x 10^9^/L
August 17, 2021	325 x 10^9^/L
September 25, 2022	279 x 10^9^/L
July 10, 2023	13 x 10^9^/L
Reference value	150-450 x 10^9^/L

**Figure 1:**
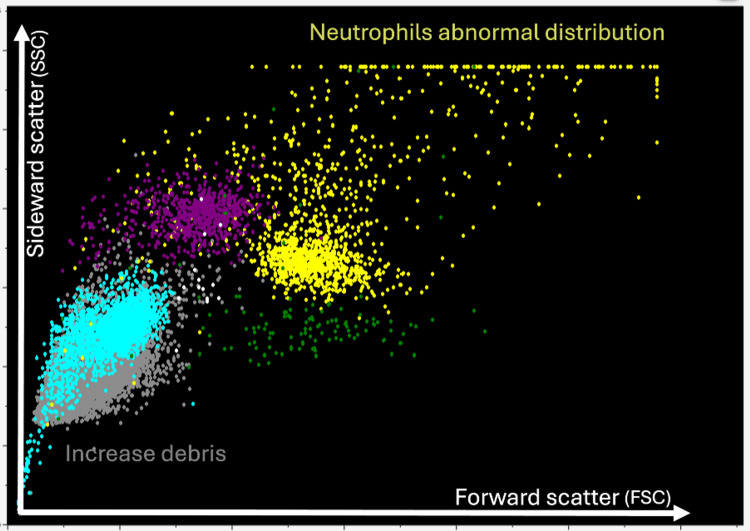
Neutrophil abnormal distribution on the histogram. Abnormal plot areas of the patient’s histogram from his peripheral blood sample. The extreme upper-right corner represents neutrophil clumping based on density and cell size, while the extreme lower-left corner indicates increased sample debris due to cell clumping and cold agglutination.

**Figure 2:**
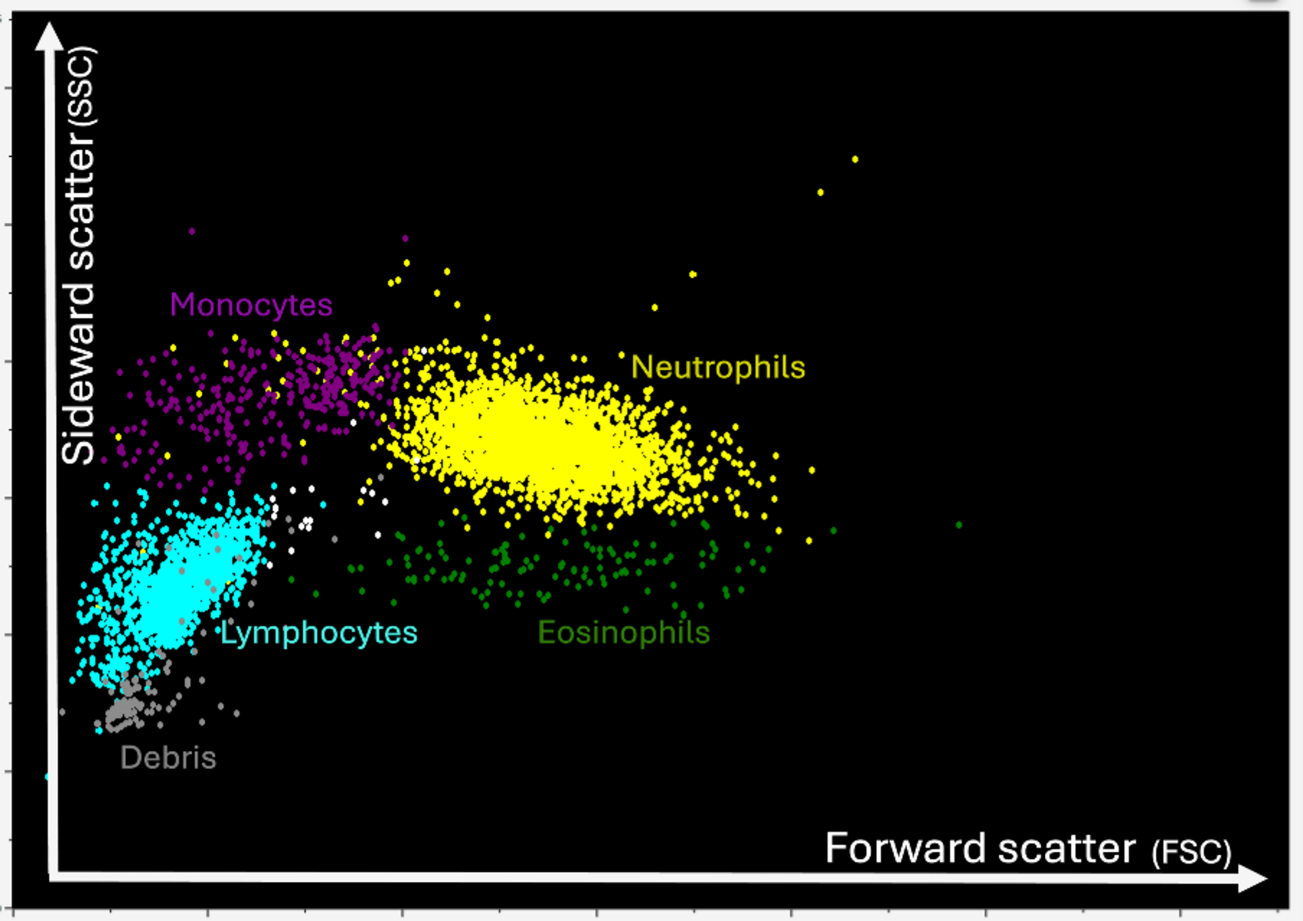
Normal donor WBC histogram. Normal WBC plot areas on a histogram from a peripheral blood sample. The side scatter measurement provides information about the internal complexity (i.e., granularity) of a cell, while the forward scatter measures relative cell size. WBC, white blood cell

The patient was again treated with prednisolone (60 mg for five days, then tapered by 5 mg every three days), along with rituximab (375 mg weekly for four doses). Following this treatment, the patient’s platelet count remained stable for nearly a year. Then, in July 2023, the patient returned for a routine checkup, and his CBC revealed a critically low platelet count (PLT: 13 × 10⁹/L) and a low neutrophil count (Neu: 1.32 × 10³/L), while his white blood cell count remained normal (WBC: 6.89 × 10³/L) (Table 2). The CBC histogram revealed an abnormal plot in the neutrophil and debris areas (Figure 1). When comparing the patient’s histogram with a normal WBC histogram from a donor peripheral blood sample, an atypical cell distribution was observed (Figure 2).

**Table 2:** Patient CBC at the time of pseudo-neutropenia detection. CBC, complete blood count; WBC, white blood cell count; Neu, neutrophil count; Lym, lymphocyte count; Mono, monocyte count; Eos, eosinophil count; Baso, basophil count; RBC, red blood cell count; HGB, hemoglobin; HCT, hematocrit; MCV, mean corpuscular volume; MCH, mean corpuscular hemoglobin; MCHC, mean corpuscular hemoglobin concentration; RDW, red cell distribution width; PLT, platelet count; MPV, mean platelet volume.

CBC	Results	Reference range
WBC	6.89 x 10^3^/L	4.00-11.0
Neu	1.32 x 10^3^/L	2.00-8.00
Lym	3.7 x 10^3^/L	1.00-4.40
Mono	1.09 x 10^3^/L	0.50-1.10
Eos	0.175 x 10^3^/L	0.10-0.70
Baso	0.02 x 10^3^/L	0.0-0.10
RBC	4.13 x 10^6^/L	4.50-6.10
HGB	111 g/L	120-180
HCT	0348 L/L	0.36-0.54
MCV	84.3 fL	76.0-97.0
MCH	26.8 pg	27.0-32.0
MCHC	318 g/L	320-350
RDW	14.60%	11.5-14.5
PLT	13 x 10^9^/L	150-450
MPV	15.20%	8.90-12.8

**Figure 3:**
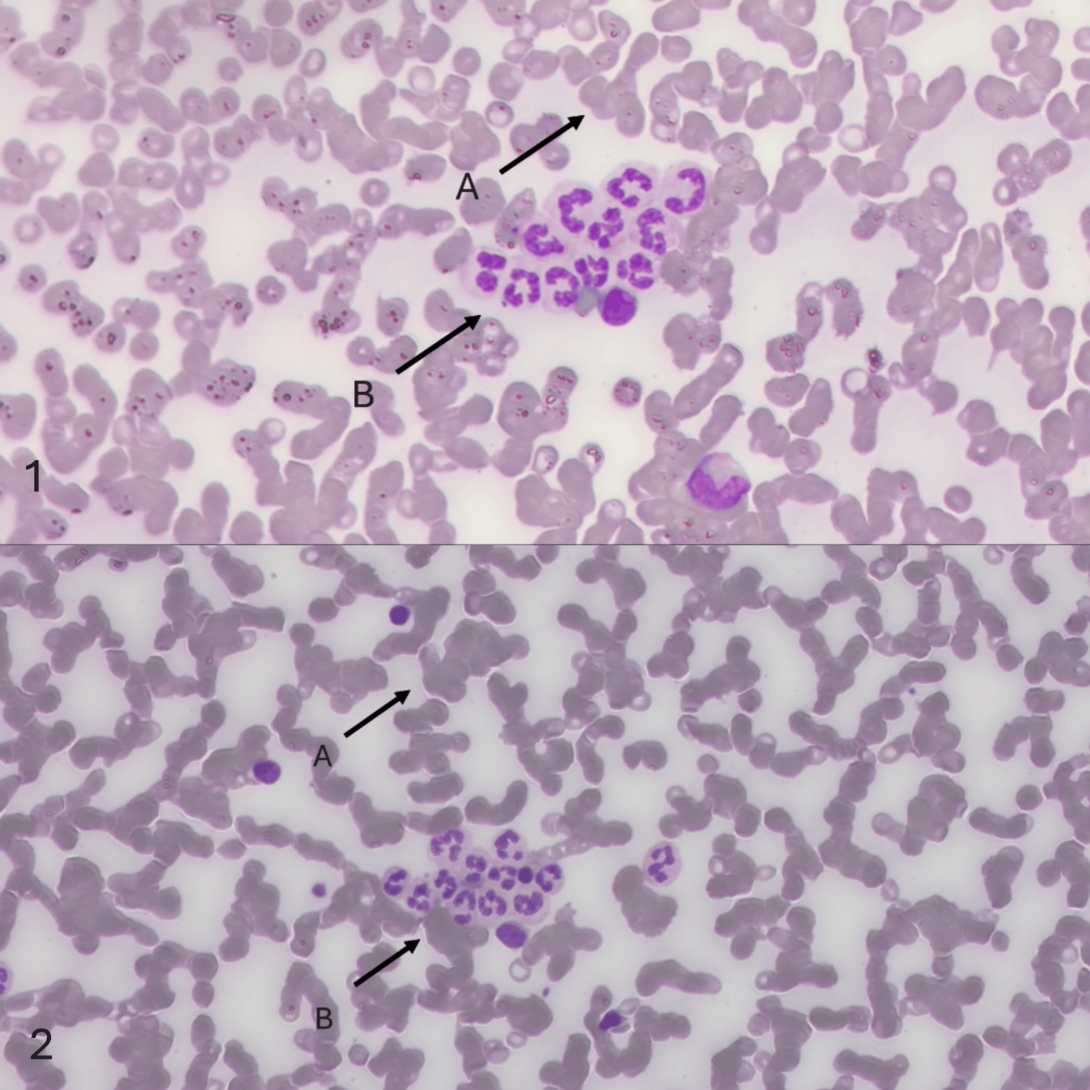
Images 1 and 2, captured using a digital microscope, show the patient’s peripheral blood smear. In both images 1 and 2, A shows red blood cells with cold agglutination, while B shows neutrophil clumping. No evidence of platelet clumping was observed, indicating true thrombocytopenia.

Then, we performed a blood film to check for platelet clumping, but none was observed. However, a new phenomenon was discovered - smear examination revealed red blood cells with moderate cold agglutination and white blood cells with neutrophil clumping (Figure 3). When a manual differential was performed, it confirmed that neutrophil clumping caused pseudo-neutropenia. The patient was treated with prednisolone (1 mg/kg, tapered by 5 mg every three days until discontinued), lifelong eltrombopag (50 mg), a proton pump inhibitor (PPI), vitamin D, and calcium.

## Discussion

Pseudo-thrombocytopenia, or spurious thrombocytopenia, refers to in vitro changes to the platelets. It occurs when the patient’s blood is sensitive to the anticoagulant media in the EDTA tube, causing the platelets in the sample to clump [3]. Neutrophil aggregation resulting in spurious neutropenia is a rare phenomenon, often leading to inaccurate interpretation of WBC counts in laboratory settings [4-7]. Similar cases have been documented, although they remain infrequent [10-12]. In this case, the patient’s history of ITP relapse compounded the diagnostic challenge, as hematological irregularities were incorrectly attributed to the underlying disease [8]. A comprehensive evaluation, including peripheral blood film examination, was essential in identifying the in vitro nature of neutrophil aggregation and distinguishing it from true neutropenia [9].

## Conclusions

Our patient had chronic ITP, which appeared under the microscope as PLT clumps on one occasion and neutrophil clumps on another. In addition to the need for continued monitoring of the relationship between ITP and cell aggregations in vitro, this case report suggests a supplementary pathway for ITP and the autoimmune activation process in vitro.
